# Antibacterial Activity of Three Extra Virgin Olive Oils of the Campania Region, Southern Italy, Related to Their Polyphenol Content and Composition

**DOI:** 10.3390/microorganisms7090321

**Published:** 2019-09-05

**Authors:** Filomena Nazzaro, Florinda Fratianni, Rosaria Cozzolino, Antonella Martignetti, Livia Malorni, Vincenzo De Feo, Adriano G. Cruz, Antonio d’Acierno

**Affiliations:** 1 Istituto di Scienze dell’Alimentazione-Consiglio Nazionale delle Ricerche (CNR-ISA), Via Roma 64, 83100 Avellino, Italy; 2 Dipartimento di Farmacia, Università di Salerno, Via Giovanni Paolo II, 132, Fisciano, 84084 Salerno, Italy; 3 Instituto Federal de Educação, Ciência e Tecnologia di Rio de Janeiro (IFRJ), Departamento de Alimentos, Rio de Janeiro 20270-021, Brazil

**Keywords:** extra virgin olive oil, polyphenols, antimicrobial activity

## Abstract

Production of extra virgin olive oil (EVOO) represents an important element for the economy of Southern Italy. Therefore, EVOO is recognized as a food with noticeable biological effects. Our study aimed to evaluate the antimicrobial activity exhibited by the polyphenolic extracts of EVOOs, obtained from three varieties of *Olea europea* L. (*Ruvea antica*, *Ravece*, and *Ogliarola*) cultivated in the village of Montella, Avellino, Southern Italy. The study evaluated the inhibiting effect of the extracts against some Gram-positive and Gram-negative bacteria. Statistical analysis, used to relate values of antimicrobial activity to total polyphenols and phenolic composition, revealed a different behavior among the three EVOO polyphenol extracts. The method applied could be useful to predict the influence of singular metabolites on the antimicrobial activity.

## 1. Introduction

Extra virgin olive oil (EVOO) is a food extracted by the mechanical pressing of the fruits of the olive tree (*Olea europaea* L.). EVOO and other products from olive tree are central components of the Mediterranean diet, characterized, as it is well known, by a scarce intake of products of terrestrial animal origin, and, concomitantly, by a high intake of fruits, vegetables, cereals, fish, as well as by a moderate wine consumption. Fruits and vegetables, including cereals, are rich in phytochemicals, with proven protective effects in limiting several chronic diseases, such as cancer and cardiovascular illnesses. EVOO represents an important source of nutritionally and healthfully compounds, so that it is considered as a real functional food [1]. Apart from fatty acids (mainly triglycerides, fat-soluble substances and polar compounds, representing 95–98% of the whole EVOO)—pulp and seed of olive contain several other types of compounds, which are present in the final product after the extractive process. Polyphenols are probably one of the most important groups of minor polar components present in the EVOO. The biological importance of polyphenols gives rise from their numerous ascertained biochemical activities, such as the prevention of oxidation reactions to fatty acids. In addition, for this reason they contribute to the stability of the oil over time, delaying rancidity. Polyphenols are also capable of preventing and inhibiting radical-type reactions in the human body, thus limiting the formation of anomalous molecules that might alter the smooth functioning of cell membranes. Generally, EVOO is rich in polyphenols, until 1 g gallic acid equivalents (GAE)/kg of product [2]. The principal subfamilies of polyphenols detectable in the EVOO are phenolic acids, phenolic alcohols, secoridoids, lignans and flavonoids. Each of the above-mentioned subfamilies can then be differentiated from the others by chemical composition and reactivity, as well as, probably, by its organoleptic characteristics. It is therefore clear that the proportions and rate between the different polyphenols present in the EVOO considerably change its nutraceutical and sensory qualities. Olives and its derived-products, including EVOO, are capable, within certain limits, to resist against the biotic and abiotic stresses, for instance against pathogen attack, affecting the host-pathogen interaction. Such property is mainly due to the presence of polyphenols, which can also exhibit antimicrobial activity [3]. Polyphenols of EVOOs are able to inhibit in vitro, generally in a synergistic way, the growth of pathogens responsible for some intestine and respiratory diseases. Olive polyphenols could contribute in inhibiting the growth of *Helicobacter pylori* [4] and that of some foodborne pathogens, such as *Escherichia coli*, *Listeria monocytogenes* and *Salmonella enteriditis* [5]. EVOO demonstrated a good antimicrobial effect against *Salmonella* Typhi [6]. EVOO polyphenols are considerably absorbed (up to 95%) in humans mainly in the small intestine, where they might exert a significant local action [7]. Therein, they undergo different fate: some of them are directly absorbed; others are metabolized giving rise to other molecules, which can play a double role: act against enteropathogens, for instance, and, among other activities, improve the growth of beneficial microbes, acting as prebiotics [8,9]. Taking also into account the bioavailability of polyphenols, several authors ascertained that the use of EVOO in food might help in supporting the prevention against foodborne pathogens [5,10]. Recently, the inhibitory effect of EVOO polyphenols was demonstrated also against some oral microorganisms, such as oral streptococci, *Porphyromonas gingivalis*, *Fusobacterium nucleatum*, and *Parvimonas micra* [11]. In olive mill wastewater, phenolic compounds and their secoiridoid derivatives present in an ethanol fraction contribute to support the noticeable antimicrobial activity exhibited against the foodborne pathogen *Campylobacter* [12]. Cultivar, genetics, agronomic practices and climatic conditions, as well as the degree of ripening, storage conditions and fruit processing techniques are all factors that may affect the characteristics of EVOO, including the polyphenol profile and the subsequent biological properties [13,14]. The aim of our work was to evaluate the antibacterial activity exhibited by the polyphenol fraction of EVOOs, produced with the fruits of three varieties of *Olea europea* L. (*Ruvea antica*, *Ravece*, and *Ogliarola*) cultivated in Southern Italy. The study evaluated in particular the inhibitory effect of the extracts against several Gram-positive and Gram-negative bacterial strains. Statistical analysis correlated the antibacterial activity to the total polyphenols and to the percentage of the single components identified by a chromatographic approach within the three extracts.

## 2. Materials and Methods

The EVVOs used in this study were obtained by cold pressing from three varieties, *Ruvea antica*, *Ogliarola*, and *Ravece* of *O. europea,* grown in the village of Montella, Irpinia province, Campania region, Southern Italy. Samples of the three varieties were identified by Vincenzo De Feo, University of Salerno. Voucher specimens of the three varieties are stored in the herbarium of the University of Salerno.

### 2.1. Polyphenols Analysis

#### 2.1.1. Standards and Reagents

Most of the standards used for the Ultra Pressure Liquid Chromatography (UPLC) analysis (caffeic, ferulic, *p*-coumaric, gallic, and chlorogenic acids; catechin; quercetin; 3-hydroxytyrosol, spiraeoside, oleureopin, dadzein, luteolin, naringenin, formononentin), as well as high pressure liquid chromatography (HPLC)-grade ethanol and acetonitrile were purchased from Sigma-Aldrich (Milano, Italy). Apigenin and hyperoside were purchased from Extrasynthese (Genay, France).

#### 2.1.2. Extraction and Determination of Total Polyphenols

The extraction of polyphenols from EVOOs, necessary for the chromatographic analyses, was performed using hexane (1:1 *w*/*v*), following the method of Fratianni et al. [15]. The mixture was then charged onto cartridges SPE C_18_, and eluted three times with methanol. The three residues were pooled, dried, re-suspended in 1 mL of methanol and filtered through a 0.20 mm filter before the analysis. Total phenolic (TP) content was determined using the Folin-Ciocalteau reagent [16]. The absorbance at λ = 760 nm was determined (Cary UV/Vis spectrophotometer, Varian, Palo Alto, CA, USA) at room temperature. A standard curve generated using gallic acid as standard was used to quantify total polyphenols.

#### 2.1.3. Chromatographic Analysis

Polyphenol composition was obtained through ultra-high-performance liquid chromatography (UPLC) using an ACQUITY Ultra Performance system linked to a PDA 2996 photodiode array detector (Waters, Milford, MA, USA), linked to an Empower software (Waters). The analysis was performed following the method of Ombra et al. [17] at λ = 280 nm with a reversed-phase column (BEH C_18_, 1.7 µm, 2.1 mm× 100 mm, Waters), at 30 °C, at a flow rate of 250 μL/min, and with pressure ranging from 6000 to 8000 psi. The effluent was introduced to an LC detector (scanning range 210–400 nm, resolution 1.2 nm). The injection volume was 5 μL. Phenolic compounds were identified and quantified through comparison of the peak areas on the chromatograms of samples with those of diluted standard solutions.

### 2.2. Antibacterial Activity

#### 2.2.1. Microorganisms and Culture Conditions

Five Gram-positive (*Bacillus cereus* DSM 4313, *Bacillus cereus* DSM 4384, *Staphylococcus aureus* DMS 25923, *Enterococcus faecalis* DSM 2352 and *Listeria innocua* DSM 20649) and two Gram-negative (*Escherichia coli* DSM 8579, and *Pseudomonas aeruginosa* ATCC 50071) bacterial strains were cultured for 18 h in Luria Bertani (LB) broth (Sigma, Milano, Italy) at 37 °C and 80 rpm (Corning LSE, Pisa, Italy).

#### 2.2.2. Determination of the Antibacterial Susceptibility by Agar Diffusion

The agar diffusion test was performed following the method of Fratianni et al. [18] with some modifications. Microbial suspensions (1 × 10^7^ colony-forming units (cfu)/mL) were spread on LB agar plates in sterile conditions. Different amounts of extracts (2.5 and 4.9 µg) were spotted on the inoculated plates. After 10 min in sterile conditions, plates were incubated at 37 °C for 24 h. The diameter of the clear zone shown on plates (inhibition zone) was accurately measured (“Extra steel Caliper mod 0289”, mm/inch reading scale, precision 0.05 mm, Mario De Maio, Milan, Italy). Sterile dimethylsulfoxide (DMSO, Sigma Aldrich Italy, Milano, Italy) and tetracycline (7 µg; Sigma Aldrich Italy) served as the negative and positive control, respectively. The experiments were performed in triplicate and averaged.

#### 2.2.3. Minimal Inhibitory Concentration (MIC)

The resazurin microtiter-plate assay [19] was used to evaluate the MIC. Samples were dissolved in sterile DMSO; then, they were distributed in a multiwell plate with different volumes of sterile Muller-Hinton broth (Sigma Aldrich Italy) previously prepared. Two-fold serial dilutions were performed to have 50 μL of the test material in serially descending concentrations in each well. A 35 μL amount of 3.3 × strength iso-sensitized broth and 5 μL of resazurin, used as indicator solution, were added to achieve a final volume/well of 240 μL. Finally, 10 μL of bacterial suspension was added to each well to reach a concentration of about 5 × 10^5^ cfu/mL. Sterile DMSO and ciprofloxacin (Sigma Aldrich Italy, prepared dissolving 1 mg/mL in DMSO) were used as negative and positive control, respectively. Multiwell plates were prepared in triplicate and incubated at 37 °C for 24 h. The lowest concentration at which a color change occurred (from dark purple to colorless) revealed the MIC value.

### 2.3. Statistical Analysis

Data were expressed as mean ± standard deviation of triplicate measurements. The PC software “Excel Statistics” was used for the calculations. The analysis correlated the values of antibacterial activity, specifically to the inhibition zone data, to total polyphenols and phenolic composition, using the free software environment for statistical computing and graphics R (https://www.r-project.org/) [15].

## 3. Results and discussion

### 3.1. Antibacterial Activity of the Extracts

The antibacterial capability of the polyphenol (PF) extracts of *Ogliarola*, *Ravece*, and *Ruvea antica* EVOOs was assayed against different Gram-positive and Gram-negative bacteria, through the inhibition zone test and the determination of the Minimal Inhibitory Concentration (MIC). Results are shown in Table 1 and Table 2 respectively.

The minimum concentration necessary to inhibit the growth of the pathogenic tester strains was low for all the PF extracts, usually equal to 1–2 μg, except when PF of *Ravece* were tested against *S. aureus* (MIC > 15 μg), and when those of *Ruvea antica* were assayed against *E. faecalis* (MIC > 10 μg). This confirms that polyphenols present in the EVOO have a general capacity to inhibit the growth of pathogenic or unwanted microorganisms [3]. Therefore, different in vitro studies demonstrated that some polyphenols from olive oil are able to inhibit the growth of different bacteria, including those responsible for some respiratory infection and intestinal diseases, as well as against bacteria, such as *Helicobacter pylori*, one of the agents of peptic ulcers and some types of cancer [4,20].

In general, 4.9 μg of the PF extract from *Ogliarola* were very effective in inhibiting the microbial growth of all the strains considered, with inhibition zone not lesser than 10.67 (against *L. innocua*) up to 18.33 mm (against *B. cereus* 4313). Overall, 4.9 μg of the polyphenol extract from *Ravece* produced inhibition zones also superior to 17 mm (17.33 mm, against *B. cereus* 4313 and *E. faecalis*). 4.9 μg of PF extract from *Ruvea antica* resulted less effective, producing zones not greater than 12.67 mm. All three EVOO PF extracts were effective in inhibiting the growth of *E. coli*, producing (with 4.9 μg of PF extracts from *Ravece* and *Ogliarola*) inhibition zones up to 13 mm. This result, in our opinion, could find an interesting practical application. *E. coli* is the most frequent cause of urinary tract infections. Like other *E. coli* pathotypes, the strain used in our experiments differs from the commensal *E. coli*, due to the presence of some virulence factors, which can concur, with other microbial systems, to increase its resistance against conventional antibiotics, to form biofilm, as well as to contaminate food or medical support (e.g., catheters), with difficulty to eradicate the infection and serious damage to health [21]. Thus, the capability of EVOO polyphenols to avoid the growth of this pathogen strain could be exploited not only for the EVOO per se, or for the great bioavailability of EVOO PFs, but also taking into account that the EVOO by-products are rich in polyphenols, which can convert them from a problem for the environment to a resource of biomolecules of high added value, potentially useful for food and pharmaceutical purposes. Therefore, other olive by-products, such as leaves demonstrated activity against different species of pathogens, including those used in our experiments [22]. The three PF extracts were also capable of inhibiting the growth of *Ps. aeruginosa*. Such microorganism, similar to *E. coli*, not only is a well-known pathogen, but it is also capable to form biofilm, increasing its resistance to the conventional drugs [23]. The effect was well visible, so that we measured inhibition halos until 8.67 mm just using 2.5 μg. In both cases, the extracts *Ogliarola* and *Ravece* were more effective than those of *Ruvea antica* in inhibiting the growth of the strain; in particular, 2.5 μg of PF extract of *Ravece* were twice as effective as that of *Ruvea antica* against *Ps. aeruginosa*; 4.9 μg of *Ravece* PF extracts were even three times more effective than the *Ruvea antica* ones. The different effectiveness exhibited by the extracts against the two strains of *B. cereus* (DSM 4313 and DSM 4384) proved once again that the resistance/sensitivity of a microorganism to a natural extract or to a singular compound might be not only linked to the genera or species but, in some cases, it might even be strain-specific [24,25].

### 3.2. Statistical Analysis

Some of the individual phenolic compounds present in the EVOOs extracts were identified and quantified by UPLC. However, the choice to evaluate the antibacterial activity of the entire extracts was taken for different reasons. First, the antibacterial activity of phenolic compounds is generally well-known [26,27,28,29,30,31]. Moreover, PF extracts might exhibit more beneficial effects than their individual constituents, which can change own properties in the presence of other compounds present in the extracts [32]. As said by Liu [33], the health benefits of fruits and vegetables give rise from synergistic effects of phytochemicals and the advantages on human health of a diet rich in fruits and vegetables is attributed to the complex mixture of phytochemicals present in whole foods. This explains why generally no individual antibacterial effect can substitute the combination of natural phytochemicals to achieve the health benefits [34]. Thus, we statistically correlated the total polyphenols and individual molecules to the antibacterial activity exhibited by the EVOO extracts. The correlation between total polyphenols and the average antibacterial activity resulted high (=0.85). We identified 10 polyphenols through UPLC analysis, based on the retention time of corresponding standards. For all of them, we calculated the percentage present in each extract. Data on polyphenol composition are reported in Table 3. The statistical approach allowed us to divide such molecules into different groups, with respect to their potential influence on the average antibacterial activity of the extracts. Correlation coefficients (Corr-coeffs) are reported in Table 4. In the first group, we found that flavonol quercetin and isoflavone formononetin, which Corr-coeffs (0.94 and 0.97, respectively) seemed to let us foresee by the whole their highest influence on the antibacterial activity with respect to the other molecules. Other two polyphenols, flavanone naringenin and the secoiridoid oleuropein exhibited lower Corr-coeffs (0.55 and 0.47, respectively).

**Figure 1:**
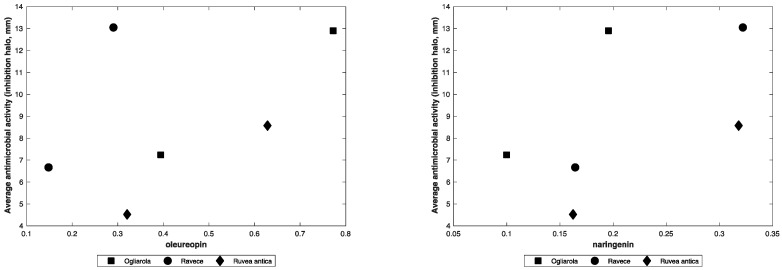
Average antibacterial activity exerted by the three PF extracts vs. oleuropein (**left**) and vs. naringenin (**right**). On X it is reported the amount (in µg) of the molecules present in 2.5 and 4.9 µg of the PF extracts tested.

Taking into account the percentage of the two molecules in the extracts, it is possible to hypothesize for this other group a little bit of predominance of correlation between oleuropein and the average antibacterial activity of the ‘Ravece’ extract (Figure 1, left) and between naringenin on the average antibacterial activity exerted by the ‘Ogliarola’ extract (Figure 1, right).

The correlation between another group of polyphenols and the antibacterial activity of the extracts was still less strict; thus, flavone luteolin (Corr-coeff = 0.37) and the hydroxycinnamic *p*-coumaric acid (Corr-coeff = 0.33) seemed to break the antibacterial activity of the extract *Ogliarola*. Concurrently, isoflavone dadzein (Corr-coeff = 0.28) and flavonol spiraeoside (Corr-coeff = 0.27) did not seem to enhance that of the extract *Ravece*. The other flavone apigenin exhibited a negative coefficient of correlation (Corr-coeff = −0.34). This metabolite is a known antibacterial compound [34,35]. However, in some cases its effect could be nil against some microorganisms [36].

The statistical approach was also applied to evaluate the correlation between the singular molecules and the antibacterial activity with respect to the microorganisms. Table 5 reports the coefficients of correlation.

With respect to the strains used in the agar diffusion test, we could suppose a noticeable inhibitory effect of formononentin and quercetin against *B. cereus*. In fact, both strains of *B. cereus* (DSM 4313 and DSM 4384) seemed to be strongly inhibited by the presence of these two metabolites (Corr-coeffs = 0.97 and 0.95, respectively); concurrently, quercetin seemed to prevent the bacterial growth too (Corr-coeffs = 0.96 and 0.93, respectively). A similar effect was hypothesized against *E. coli* (Corr-coeffs = 0.94 and 0.90, respectively) and against *E. faecalis* (Corr-coeffs = 0.91 and 0.75, respectively). Thus, for instance, if formononentin seemed to confirm its influence also against *Ps. aeruginosa* (Corr-coeff = 0.95) and *L. innocua* (Corr-coeff = 0.91), on the other hand the effect of quercetin versus these two microorganisms seemed to be less effective (Corr-coeffs = 0.74 and 0.77, respectively). Therefore, other studies demonstrated a limited inhibitory effect of quercetin against *Ps. aeruginosa* [37]. A potential inhibitory effect exhibited also by luteolin (Corr-coeff = 0.73) and *p*-coumaric acid (Corr-coeff = 0.66) against *Ps. aeruginosa* was observed indeed. At the same time naringenin (Corr-coeff = 0.78), luteolin (Corr-coeff = 0.59) and *p*-coumaric acid (Corr-coeff = 0.58) would concur in influencing, although with minor efficacy, the potential antibacterial activity of the extracts against *L. innocua*. The potential behavior exhibited by metabolites on the antibacterial activity-hypothesized through such approach- seemed to be completely different when we considered *S. aureus*. In fact, by the analysis of correlation coefficients we could hypothesize that other metabolites in place of formononentin and quercetin may have contributed to the antibacterial activity of the extracts, in particular spiraeoside, dadzein, and catechin (Corr-coeffs = 0.91; 0.90 and 0.80, respectively). Moreover, this was the unique case in which oleuropein (one of the most important and known metabolites characterizing the EVOO polyphenols) seemed to have contributed to the antibacterial activity of the extracts (Corr-coeff = 0.89). Therefore, oleuropein as well as 3-hydroxytirosol (which in our case showed a correlation coefficient of 0.84) have antibacterial activity against *S. aureus*, as demonstrated by Bisignano et al. [38]. Concurrently, statistics confirmed the controversial behavior exhibited by 3-hydroxytirosol that was active against *S. aureus* but had lower effect (Corr-coeff = 0.47) against *E. coli*, corroborating the indications given by other studies [39]. The fact that the *Ravece* extract did not contain dadzein might suggest that such metabolite in particular affected the resistance of *S. aureus*. In fact, as shown in Table 2, the MIC *Ravece* extract *versus S. aureus* was higher than 10 µg and much lower in the case of the other two extracts. The absence of catechin, which gave a correlation coefficient of 0.80 and the concurrent presence of luteolin (6.22% in *Ravece*, Corr-coeff = −0.76) could have contributed to its higher MIC value. Concomitantly, the presence of apigenin found only in the *Ruvea antica* extract with the most negative coefficient of correlation (= −0.75) would seem to support its influence on the resistance of *E. faecalis* versus that extract, as indicated by the MIC value and by the results of the inhibition zone test.

## 4. Conclusions

The polyphenol fraction present in EVOO oil confirms once again its antibacterial properties. The different qualitative and quantitative profile of polyphenols present in a PF extract can affect in a different way its antibacterial effectiveness. The statistical method herein applied is easy and useful to predict the synergistic effect of polyphenols and the influence that each of them has—based also on their amount—on the activity of the whole extract. In a future perspective this could be a basis of possible complementary studies, for example, to formulate ideal drugs of natural origin, composed of optimal mixtures of polyphenols which are able to exercise with the minimum effort (in terms of quantity) and the maximum result (against the greatest number of pathogens) their antibacterial efficacy.

## Figures and Tables

**Table 1:** Antibacterial activity evaluated through the inhibition zone test of the three polyphenol (PF) extracts of *Ogliariola*, *Ravece* and *Ruvea antica* EVOOs, against different pathogens. The test was performed using 2.5 and 4.9 µg of extract. Data are expressed in mm. Results are shown as mean (± SD) (*n* = 3). For details, see Materials and Methods.

	‘Ogliarola’		‘Ravece’		‘Ruvea Antica’		Tetracycline
	2.5 µg	4.9 µg	2.5 µg	4.9 µg	2.5 µg	4.9 µg	7 µg
*E. coli*	7.30 (±0.57)	13.30 (±0.57)	7.00 (±0.57)	13.67 (±0.28)	5.30 (±0.52)	10.00 (±0.00)	12.67 (±0.57)
*L. innocua*	5.67 (±0.57)	10.67 (±0.57)	6.67 (0.57)	13.33 (±0.57)	4.30 (±0.57)	9.30 (±0.57)	10.33 (±0.50)
*S. aureus*	7.30 (±0.57)	11.67 (±0.57)	0.00 (±0.00)	0.00 (±0.00)	6.67 (±0.57)	12.67 (±0.57)	6.67 (±0.57)
*B. cereus 4313*	10.67 (±1.14)	18.33 (±0.57)	9.67 (±0.57)	17.33 (±1.15)	6.33 (±0.57)	11.67 (±0.57)	9.67 (±0.57)
*B. cereus 4384*	7.67 (±0.57)	13.67 (±0.57)	7.67 (±0.57)	17.30 (±1.14)	0.00 (±0.00)	0.00 (±0.00)	8.30 (±1.05)
*P. aeruginosa*	6.33 (±0.57)	11.33 (±0.57)	8.67 (±0.57)	16.33 (±0.57)	4.33 (0.57)	6.67 (±0.57)	10.00 (±0.00)
*E. faecalis*	5.67 (±0.57)	11.33 (±0.57)	7.67 (±0.57)	17.33 (±1.14)	0 00 (±0.00)	0.00 (±0.00)	12.33 (±0.57)

**Table 2:** Minimal Inhibitory Concentration (MIC, μg/mL) of the PF extracts of ‘Ogliarola’, ‘Ravece’ and ‘Ruvea antica’ EVOOs, evaluated through the resazurin test, as reported in the Materials and Methods section.

	*Ogliarola*	*Ravece*	*Ruvea Antica*
*B. cereus* 4313	1.00	1.00	1.00
*B. cereus* 4384	1.00	1.00	2.00
*E.coli*	1.00	1.00	2.00
*P. aeruginosa*	1.00	1.00	2.00
*S. aureus*	1.00	>15.00	2.00
*L. innocua*	2.00	2.00	2.00
*E. faecalis*	2.00	2.00	>10.00

**Table 3:** Polyphenol composition, obtained by Ultra Pressure Liquid Chromatography (UPLC), of the three PF extracts of *Ogliarola*, *Ravece* and *Ruvea antica* EVOOs. The data are reported as percentage of total polyphenols.

Polyphenols (%)	‘Ogliarola’	‘Ravece’	‘Ruvea Antica’
Gallic acid	0.00	0.00	0.00
3 Hydroxytirosol	1.86	0.43	1.10
Catechin	1.08	0.00	0.43
*p*-Coumaric acid	0.00	0.28	0.11
Quercetin-4-glucoside (spiraeoside)	9.48	0.00	5.75
Oleuropein	15.77	5.92	12.82
Dadzein	4.13	0.00	2.36
Luteolin	0.00	6.22	1.57
Quercetin	24.06	18.03	10.61
Apigenin	0.00	0.00	3.18
Naringenin	3.99	6.57	6.49
Formononentin	4.45	4.81	2.27

**Table 4:** Correlation coefficients between the potential average antibacterial activity and polyphenols identified in the extracts of *Ogliarola*, *Ravece* and *Ruvea antica* EVOOs. The analysis was elaborated with respect to the percentage of each molecule present in the extracts and in an independent way with respect to the pathogens.

Polyphenols	Corr-Values
Formononentin	0.97
Quercetin	0.94
Naringenin	0.55
Oleuropein	0.47
Luteolin	0.37
Catechin	0.35
*p*-Coumaric acid	0.33
Dadzein	0.28
Spiraeoside	0.27
Apigenin	−0.34

**Table 5:** Correlation coefficients between the potential antibacterial activity and polyphenols identified in the extracts of ‘Ogliarola’, ‘Ravece’ and ‘Ruvea antica’ EVOOs, with respect to different pathogens. The analysis was elaborated with respect to the percentage of each molecule present in the extracts, taking into account the amounts (2.5 µg and 4.9 µg) of the extracts used to determine the antibacterial activity of the extracts against different pathogens. BC: *Bacillus cereus* (strains DSM 4313 and DSM 4384); EC: *Escherichia coli*; LI: EF: *Enterococcus faecalis*; *Listeria innocua*; SA: *Staphylococcus aureus;* PA: *Pseudomonas aeruginosa*.

	Microorganisms						
Polyphenol	BC 4313	BC 4384	EC	EF	LI	SA	PA
Formononentin	0.97	0.95	0.94	0.91	0.91	−0.16	0.95
Quercetin	0.96	0.93	0.90	0.75	0.77	0.18	0.74
Naringenin	0.47	0.57	0.65	0.26	0.78	0.02	0.55
Oleuropein	0.50	0.53	0.51	−0.09	0.33	0.89	0.00
Luteolin	0.30	0.33	0.39	0.62	0.59	−0.76	0.73
Catechin	0.41	0.38	0.33	−0.04	0.086	0.80	−0.10
*p*-Coumaric acid	0.25	0.30	0.36	0.52	0.58	−0.69	0.66
Dadzein	0.34	0.33	0.29	−0.19	0.06	0.90	−0.19
Spiraeoside	0.32	0.32	0.27	−0.21	0.05	0.91	−0.21
Apigenin	−0.38	−0.27	−0.21	−0.75	−0.15	0.56	−0.51
3-Hydroxytyrosol	0.51	0.51	0.47	−0.01	0.25	0.84	0.00

## References

[1] Ray N.B., Hilsabeck K.D., Karagiannis T.C., McCord D.E., Singh R.B., Watson R.R., Takahashi T. (2019). Bioactive Olive Oil Polyphenols in the Promotion of Health. The Role of Functional Food Security in Global Health.

[2] Gorzynik-Debicka M., Przychodzen P., Cappello F., Kuban-Jankowska A., Marino Gammazza A., Knap N., Wozniak M., Gorska-Ponikowska M. (2018). Potential health benefits of olive oil and plant polyphenols. Int. J. Mol. Sci..

[3] Capasso R., Evidente A., Schivo L., Orru G., Marcialis M.A., Cristinzio G. (1995). Antibacterial polyphenols from olive oil mill waste waters. J. Appl. Bacteriol..

[4] Romero C., Medina E., Vargas J., Brenes M., De Castro A. (2007). In vitro activity of olive oil polyphenols against *Helicobacter pylori*. J. Agric Food Chem..

[5] Karaosmanoglu H., Soyer F., Ozen B., Tokatli F. (2010). Antimicrobial and antioxidant activities of Turkish extra virgin olive oils. J. Agric. Food Chem..

[6] Gabriel P.O., Aribisala J.O., Oladunmoye M.K., Arogunjo A.O., Ajayi-Moses O.B. (2019). Therapeutic effect of goya extra virgin olive oil in albino rat oro-gastrically dosed with *Salmonella* Typhi. South Asian J. Res. Microbiol..

[7] Rubio L., Macia A., Castell-Auvi A., Pinent M., Blay M.T., Ardevol A., Romero M.P., Motilva M.J. (2014). Effect of the co-occurring olive oil and thyme extracts on the phenolic bioaccessibility and bioavailability assessed by in vitro digestion and cell models. Food Chem..

[8] Deiana M., Serra G., Corona G. (2018). Modulation of intestinal epithelium homeostasis by extra virgin olive oil phenolic compounds. Food Funct..

[9] Nazzaro F., Fratianni F., d’Acierno A., Coppola R., Ravishankar Rai V. (2015). Gut Microbiota and Polyphenols: A Strict Connection Enhancing Human Health. Advances in Food Biotechnology.

[10] Cicerale S., Lucas L.J., Keast R.S.J. (2012). Antimicrobial, antioxidant and anti-inflammatory phenolic activities in extra virgin olive oil. Curr. Opin. Biotechn..

[11] Karygianni L., Cecere M., Argyropoulou A., Hellwig E., Skaltsounis A.L., Wittmer A., Tchorz J.P., Al-Ahmad A. (2019). Compounds from *Olea europaea* and *Pistacia lentiscus* inhibit oral microbial growth. BMC Compl. Altern. Med..

[12] Manuel Silvana J., Pinto-Bustillos M.A., Vásquez-Ponce P., Prodanov M., Martinez-Rodriguez A.J. (2019). Olive mill wastewater as a potential source of antibacterial and anti-inflammatory compounds against the food-borne pathogen *Campylobacter*. Inn. Food Sci. Em. Technol..

[13] Lazzez A., Perri E., Caravita M.A., Khlif M., Cossentini M. (2008). Influence of olive maturity stage and geographical origin on some minor components in virgin olive oil of the Chemlali variety. J. Agric. Food Chem..

[14] Rotondi A., Bendini A., Cerretani L., Mari M., Lercker G., Toschi T.G. (2004). Effect of olive ripening degree on the oxidative stability and organoleptic properties of cv. Nostrana di Brisighella extra virgin olive oil. J. Agric. Food Chem..

[15] Fratianni F., Cozzolino R., Martignetti A., Malorni L., d’Acierno A., De Feo V., Cruz A.G., Nazzaro F. (2019). Biochemical composition and antioxidant activity of three extra virgin olive oils from the Irpinia province, Southern Italy. Food Sci. Nutr..

[16] Singleton V.L., Rossi J.A. (1965). Colorimetry of total phenolics with phosphomolybdic-phosphotungstic acid reagents. Am. J. Enol. Vitic..

[17] Ombra M., d’Acierno A., Nazzaro F., Riccardi R., Spigno P., Zaccardelli M., Pane C., Maione M., Fratianni F. (2016). Phenolic composition and antioxidant and antiproliferative activities of the extracts of twelve common bean (*Phaseolus vulgaris* L.) endemic ecotypes of Southern Italy before and after cooking. Oxid. Med. Cell Longev..

[18] Fratianni F., Ombra M.N., Cozzolino A., Riccardi R., Spigno P., Tremonte P., Coppola R., Nazzaro F. (2016). Phenolic constituents, antioxidant, antimicrobial and anti-proliferative activities of different endemic Italian varieties of garlic (*Allium sativum* L.). J. Funct. Foods.

[19] Sarker S.D., Nahar L., Kumarasamy Y. (2007). Microtitre plate-based antibacterial assay incorporating resazurin as an indicator of cell growth, and its application in the in vitro antibacterial screening of phytochemicals. Methods.

[20] Medina E., de Castro A., Romero C., Brenes M. (2006). Comparison of the concentrations of phenolic compounds in olive oils and other plant oils: Correlation with antimicrobial activity. J. Agric. Food Chem..

[21] Nazzaro F., Fratianni F., d’Acierno A., De Feo V., Ayala Zavala F.J., Cruz A.G., Granato D., Coppola R., Tommonaro G. (2019). Effect of Polyphenols on Microbial Cell-Cell Communications. Quorum Sensing.

[22] Sudjana A.N., D’Orazio C., Ryan V., Rasool N., Ng J., Islam N., Riley T.V., Hammer K.A. (2009). Antimicrobial activity of commercial *Olea europaea* (olive) leaf extract. Int. J. Antimicrob. Agents.

[23] Nazzaro F., Fratianni F., Coppola R. (2013). Quorum sensing and phytochemicals. Int. J. Mol. Sci..

[24] Cerulli A., Lauro G., Masullo M., Cantone V., Olas B., Kontek B., Nazzaro F., Bifulco G., Piacente S. (2017). Cyclic diarylheptanoids from *Corylus avellana* green leafy covers: Determination of their absolute configurations and evaluation of their antioxidant and antimicrobial activities. J. Nat. Prod..

[25] Ruparelia J.P., Chatterjee A.K., Duttagupta S.P., Mukherji S. (2008). Strain specificity in antimicrobial activity of silver and copper nanoparticles. Acta Biomat..

[26] Pereira J.A., Pereira A.P.G., Ferreira I.C.F.R., Valentão P., Andrade P.B., Seabra R., Estevinho L., Bento A. (2006). Table olives from Portugal: Phenolic compounds, antioxidant potential and antimicrobial activity. J. Agric. Food Chem..

[27] Proestos C., Chorianopoulos N., Nychas G.J.E., Komaitis M. (2005). RP-HPLC analysis of the phenolic compounds of plant extracts. Investigation of their antioxidant capacity and antimicrobial activity. J. Agric. Food Chem..

[28] Rauha J.P., Remes S., Heinonen M., Hopia A., Kähkönen M., Kujala T., Pihlaja K., Vuorela H., Vuorela P. (2000). Antimicrobial effects of Finnish plant extracts containing flavonoids and other phenolic compounds. Int. J. Food Microbiol..

[29] Zhu X., Zhang H., Lo R. (2004). Phenolic compounds from the leaf extract of artichoke (*Cynara scolymus* L.) and their antimicrobial activities. J. Agric. Food Chem..

[30] Puupponen-Pimia R., Nohynek L., Meier C., Kähkönen M., Heinonen M., Hopia A., Oksman-Caldentey K.-M. (2001). Antimicrobial properties of phenolic compounds from berries. J. Appl. Microbiol..

[31] Pereira A.P., Ferreira I.C.F.R., Marcelino F., Valentão P., Andrade P.B., Seabra R., Estevinho L., Bento A., Pereira J.A. (2007). Phenolic compounds and antimicrobial activity of olive (*Olea europaea* L. Cv. Cobrançosa) leaves. Molecules.

[32] Borchers A.T., Keen C.L., Gerstiwin M.E. (2004). Mushrooms, tumors, and immunity: An update. Exp. Biol. Med..

[33] Liu R.H. (2003). Health benefits of fruits and vegetables are from additive and synergistic combination of phytochemicals. Am. J. Clin. Nutr..

[34] Cushnie T., Lamb A.J. (2005). Antimicrobial activity of flavonoids. Int. J. Antimicrob. Agents.

[35] Khanna P., Sharma O.P., Sehgal M., Bhargava C., Jain M., Goswami A., Singhvi S., Gupta U., Agarwal R., Sharma P. (1980). Antimicrobial principles from tissue culture of some plant species. Indian J. Pharm. Sci..

[36] Basile A., Sorbo S., Giordano S., Ricciardi L., Ferrara S., Montesano D., Castaldo Cobianchi R., Vuotto M.L., Ferrara L. (2000). Antibacterial and allelopathic activity of extract from *Castanea sativa* leaves. Fitoterapia.

[37] Sakharkar M.K., Jayaraman P., Soe W.M., Chow V.T.K., Sing L.C., Sakharkar K.R. (2009). In vitro combinations of antibiotics and phytochemicals against *Pseudomonas aeruginosa*. J. Microbiol. Immunol. Infect..

[38] Bisignano G., Tomaino A., Lo Cascio R., Crisafi G., Uccella N., Sajia A. (1999). On the *in-vitro* antimicrobial activity of oleuropein and hydroxytyrosol. J. Pharm. Pharmacol..

[39] Medina-Martínez M.S., Truchado P., Castro-Ibáñez I., Allende A. (2016). Antimicrobial activity of hydroxytyrosol: A current controversy. Biosci. Biotechn. Biochem..

